# Creativity and Life Satisfaction in Spanish University Students. Effects of an Emotionally Positive and Creative Program

**DOI:** 10.3389/fpsyg.2021.746154

**Published:** 2021-12-06

**Authors:** Presentación A. Caballero-García, Sara Sánchez Ruiz

**Affiliations:** Faculty of Education, Camilo José Cela University, Madrid, Spain

**Keywords:** creativity, life satisfaction, higher education, key competences, employability

## Abstract

There is an increasing demand by society that university students demonstrate competitive skills to enable them to achieve greater success when entering the workplace. Creativity and life satisfaction correlate positively with academic performance, productivity, and excellence in the working environment. The presence of creativity and emotional intelligence in the curriculum and teaching methods in Spanish universities, however, is surprisingly lacking. Studies that examine gender differences in these variables provide conflicting results. The purpose of our research is to analyse the changes produced in both creativity and life satisfaction in university students by a positive emotional and creative intervention and explore individual differences by gender. The methodology used was a quasi-experimental pre- test/post- test design with experimental/control groups. Three hundred university students (23% men and 77% women) from the Community of Madrid (Spain) completed three exercises that evaluated creativity and life satisfaction. The results show significantly higher results in creativity and life satisfaction in women, who continued to achieve high results after the intervention. Finally, we discuss the need for emotional and creative education in universities and focus on the employability and the guarantee of equal opportunities through the development of these competencies.

## Introduction

Today it is increasingly important that students leave university with the necessary skills and ability to demonstrate that they are competitive as this will enable them to achieve greater success when entering the workplace. Creativity and life satisfaction contribute to this skill, as they help nurture both personal and social development, and are positively related to academic performance (Figueiredo-Ferraz et al., 2009; Anwar et al., 2012; Belmonte, 2013; Saremi and Bahdori, 2015; Bernabé et al., 2017; Peña García et al., 2017), productivity, excellence at work (De Ávila et al., 2019) and the ability to be innovative.

The European reference framework focuses on the need for creativity to be a key aspect on which the rest of the competencies be based and emphasizes its importance in university students’ overall learning processes (Álvarez-Santullano and De Prada Creo, 2018). It has even stated that creativity is one of the educational focal points of the XXI century (Carnero Sánchez et al., 2020). Since 1996, The Cotec Foundation (Cotec Fundation, 2020) has highlighted Spain’s potential in research, development and innovation, and the prospect of this becoming Spain’s best weapon in fighting against future global shocks and achieving a balance between social well-being, prosperity and environmental sustainability.

However, it is surprising to still see the lack of inclusion of these generic or transversal competencies within the educational system (Hernández Arteaga et al., 2015), their inadequate presence in the curriculum of Spanish universities (Porto, 2008) and in teaching approaches used nowadays (Caballero-García and Sánchez, 2018; Caballero-García et al., 2019). As we move into higher education and professional life, creativity is lost, to find an orientation toward productivity (Limiñana-Gras and Javier, 2010). Cotec’s 2010 report highlighted lower levels in creativity and innovation in young Spanish university students when compared to European students (Pérez-Díaz and Rodríguez, 2010; cited by Secanella, 2016). In the same vein, studies such as those of Álvarez-Santullano and De Prada Creo (2018) evaluated the professional competencies of university students and concluded that the average across all the competencies such as technical ability, organizational skills, oral and written communication skills, initiative, motivation, and teamwork were high. The exception was creativity, hence our interest in the analysis of the lack of creativity in our university students.

Creativity is multidimensional, polysemic, and a concept that is difficult to understand (Amabile, 1983; De La Torre, 1991; Garaigordobil, 2003; Serrano, 2004). There doesn’t seem to be any single definition or single method of measurement (Elisondo and Donolo, 2011). This has resulted in the construction of different tools to assess creative capabilities, design, and creative achievements in a real context and in everyday life situations. The Creative Intelligence Test (CREA) by Corbalán et al. (2003) “is one of these valuable initiatives” (p. 78), it measures capability to be creative, divergent production, originality, and fluency and stands out for its technical and practical properties (Elisondo, 2015). As for the explanatory models of creativity, the taxonomy by antonomasia is one that focuses on creativity as a cognitive process (Tristán López and Mendoza González, 2016). Hence, our research is based on the analysis of this perspective, and the CREA test used as our measurement tool.

CREA test is theoretically based on the classic factors of creativity (divergent production, flexibility, fluency, and originality) and on the approaches of problem formulation, lateral thinking, and the study of cognitive styles (Corbalán and Limiñana Gras, 2010). It is cataloged as a cognitive measure of individual creativity according to the indicator of question generation, in the theoretical context of problem finding and problem solving, and the concept of creativity of Amabile (1983) and other authors such as Sternberg (1988) and Weisberg (1988) who define the creativity as a production of novel, useful ideas or problem solutions.

Studies by Corbalán et al. (2009) show that the CREA test is a creativity assessment instrument that shows predictive ability with statistical significance. Its statistical justification is supported, both by the size of its normative sample, close to 2,500 cases, the convergent and divergent validity indices, reliability, and other descriptive and inferential indicators; and by the adaptation of the variable’s behavior to theoretical predictions and research precedents, with respect to what is expected in relation to different classical measures of creativity. Another motive to choose this tool is due to the numerous empirical studies in which it has been used, as well as in interventions in different fields, which show its good psychometric characteristics, and the reliability and validity of its data on the creativity of the subject (see Corbalán and Limiñana Gras, 2010).

All these reasons that make the CREA test a statistically reliable and conceptually valid measure of creativity are what led us to use it as a measure of creativity in our study, in addition to its simplicity and ease of administration to the subjects in the sample.

Life satisfaction, on the other hand, is a variable characterized by its subjectivity. In our research it has been considered as a cognitive process of subjective well-being (Diener, 2000). It can be defined as a global cognitive judgment of one’s life. The evaluation carried out is not an evaluation based on objective parameters. It implies the person’s own global interpretation of his or her own happiness and life satisfaction. And, to understand it, it is necessary to consider indirect variables of a contextual or personal nature that may influence this judgment (Diener, 1994). This makes it difficult to measure, which is why it is necessary to use a method that evaluates satisfaction orientated to specific contexts and populations (Okun et al., 1984) or more than a single evaluation measure which has adequate validity and reliability (retest and convergent test). For this, our study uses the Satisfaction with Life Scale of Diener et al. (1985) and the Overall Life Satisfaction of Campbell et al. (1976), two of the most used in this area.

The Manifesto for Adult Learning in the 21st century of the European Association for The Education of Adults (European Association for the Education of Adults [EAEA], 2019) presents nine challenges to transform adult education in Europe. These include health and well-being (in which life satisfaction is highlighted for its contribution to personal development and the construction of healthier societies), people’s life skills; employment and work (where creativity is a competence that offers new employment opportunities and increases innovation and productivity at work); and social cohesion, equity, and equality; all of which are linked to the variables of our study.

Studies show that students with greater life satisfaction, are more likely to successfully complete their university studies (Figueiredo-Ferraz et al., 2009). This well-being is also linked to employability (Lantarón, 2012), as the personal skills acquired by the student are reflected favorably when presenting themselves as candidates in the labor market. Authors such as Graen (1976) and Diener and Biswas-Diener (2002) show that students with greater well-being end up performing jobs with greater responsibility and better pay. These results justify the need for an education in socio-emotional competence within a university education (Lantarón, 2014).

The implementation of initiatives to develop creativity and life satisfaction in an educational environment can be seen in action plans, programs, and initiatives, both at international and national level and show satisfactory results (Sánchez Ruiz, 2020). In relation to intervention programs carried out in university classrooms with the aim of stimulating creative thinking, there are some examples at international level such as: the “Edmodo” program, developed with students a Tabuk University, to teach mathematics (MATLAB) by developing creative, cognitive and affective skills (Goda, 2019); the program by Vally et al. (2019), which trained the creative production skills of resident students in the Arab Emirates; the “ADAPT” program developed in 1980 by Moshman, Johnston, Tomlinson-Keasey, Williams, and Eisert at Lincoln University of Nebraska; the “DOORS” program, developed in 1977 at Central Illinois University; the “COMPAS” program, carried out in seven universities by Schermerhorn, Williams and Dyckison (between 1979 and 1982); the “SOAR” program, developed at Xavier University in Louisiana (between 1977 and 1978); and the “DORIS” program (development of reasoning in science) carried out at California State University (1980). At national level, we can see examples such as The Botín Foundation (Botín Fundation, 2012) which enhance the development of creativity through art and emotions, and Allueva (2004) whose participants were students of psychopedagogy from the University of Zaragoza, where 142 interventions of creativity were carried out, obtaining medium to high differences in the pre-test/post-test results.

Programs aimed at working on life satisfaction with university students have also been shown to be effective in improving well-being and decreasing symptoms of depression, which can be deduced from the results of meta-analyses such as those of Sin and Lyubomirsky (2009). Even though the programs showed slightly improved well-being and depression, the results stabilized over a period of time (Bolier et al., 2013; Ovejero et al., 2015). Other examples of these are: the program by Remor et al. (2010); the positive psychology program by Ovejero et al. (2015); the program by Arroyo-Resino and Carrasco-Temiño (2017); and the program by Montenegro et al. (2017) amongst others.

The positive emotional and creative intervention program (EPOCREU) was implemented in this study based on a systematic review of the paper (Sánchez Ruiz, 2020). It is based on the EI ability model of Salovey and Mayer (1990); the theoretical approaches of Diener et al. (1999), Lyubomirsky and Lepper (1999), Limiñana Gras et al. (2010), and the recommendations for the development of creative thinking by authors such as Martín and Martín (2012) and Ramírez and Fuentes (2013). The intervention was designed in an *ad hoc* way, with its goal being to work on positive emotions and develop creative thinking in an integrated way in a normal classroom environment, improve the performance and competencies of emotional intelligence, improve creativity and happiness, and enhance life satisfaction of the university students who were part of the experimental group as well as to find empirical arguments to promote strategies of methodological change at the university.

Finally, studies that have analyzed gender differences in creativity offer contradictory data (Acevedo, 2012; Sánchez Ruiz, 2020). There are researchers such as Maccoby and Jacklin (1974), Wright and Stone (1998), De Zubiría et al. (2003), Chacón and Moncada (2006), Matud et al. (2007), Baer and Kaufman (2008), Limiñana-Gras and Javier (2010), Bermejo et al. (2013), and Ramírez et al. (2019) who conclude that creative potential is not influenced by the gender variable.

Other authors have found slight differences in the mean when measuring creativity by gender in favor of females, but these results have not proved to be significant (Naderi et al., 2009; Chiecher et al., 2018; Hidalgo Fuentes et al., 2018; Echegaray Antay, 2019; Barbachán Ruales et al., 2020). Finally, there are authors who have managed to confirm these differences in creativity in favor of women (Lee, 2005; Bindu and Thomas, 2006; Miranda, 2007; Limiñana-Gras and Javier, 2010; Krumm et al., 2015; Elisondo and Donolo, 2016; and Gamarra-Moscoso and Flores-Mamani, 2020, among others). Only a few research studies have found significant differences in creativity in favor of men (Zheng and Xiao, 1983; Tegano and Moran, 1989; Cox, 2003).

Some meta-analyses such as those of Baer and Kaufman (2008) or bibliographical reviews such as those of Villamizar Acevedo (2012) confirm the heterogeneity of the results obtained in gender differences in creativity research and highlight the importance of carrying out research with a sample of university students, given that studies with this type of student body in this area are scarce, hence our interest in exploring.

Regarding the gender differences in life satisfaction, as was the case with creativity, the research results are contradictory. Some authors have found significant differences in life satisfaction in favor of women in specific contexts or situations, such as in the educational field (Gilman et al., 2008; Casas et al., 2012; Cenkseven-Önder, 2012), in social relationships, the environment (Gilman et al., 2008; Chaplin, 2009; Cenkseven-Önder, 2012); and overall life satisfaction (Gilman et al., 2008; Cenkseven-Önder, 2012; Chen and Lin, 2014). Whereas men score higher on satisfaction with sport (Chaplin, 2009); health and fitness (Woynarowska et al., 2004; Bisegger et al., 2005). Nevertheless, the conclusion drawn is that the measurement of emotional aspects of life satisfaction are more sensitive to gender differences. Women show greater satisfaction when evaluated from an emotional dimension, and the results tend to be more contradictory when measured from a cognitive dimension (Costa et al., 1987; Azpiazu, 2016), which is why some authors have justified the varying range of results because of the differing methods used for measurement (Bedin and Sarriera, 2015).

Studies of gender differences in life satisfaction in university students also offer heterogeneous results, hence our interest in contrasting this circumstance in our students. For the most part, the results show the mean values in favor of women, however they do not find significant differences in life satisfaction according to gender (Cardona and Agudelo, 2007; Gavín-Chocano and Molero-López, 2019; Jurado Guillen, 2019; Motta Taminchi, 2019; Poquioma, 2019). There are few studies that corroborate the significance of such differences in favor of women (Arocena et al., 2005; Lalive et al., 2014; Al-Attiyah and Nasser, 2016; Rojas-Valverde and Fallas-Campos, 2017; Cañero et al., 2019). Some research using even smaller groups found differences in life satisfaction in favor of men (Pavot et al., 1991; Smith et al., 1999; Bernal et al., 2011; Lukaschek et al., 2017; Tomyn and Weinberg, 2018). Recent research has shown greater interest in studying this variable because of COVID-19. The results indicate that men possess greater life satisfaction than women (Vizoso, 2019; Lozano-Díaz et al., 2020).

On the basis of the foregoing, our objectives were: (1) to describe creativity and life satisfaction of our university students, (2) to analyze the changes produced in both creativity and life satisfaction by a positive emotional and creative intervention integrated into regular classroom and (3) explore individual differences by gender in these variables, in order to promote their development in both general educational system and particular university system, under conditions of equality and competence. This makes our research part of the studies that promote a change in university education toward higher quality training based on competencies for life.

## Materials and Methods

### Participants

Three hundred university students, aged between 18 and 47, from the Community of Madrid (Spain) (23% men and 77% women) participated in our study. The sample was selected through non-probability and incidental sampling type 4 (Onwuegbuzie and Collins, 2007), often used in disciplines within education (Garrett, 1953) and useful in survey research (Pathak, 2008) like ours.

Of the total sample, one hundred and sixty-two students (54% of the total amount), one hundred and twenty-two of them women (40.7%) and forty men (13.3%), were randomly assigned to the experimental group by the researchers. The control group was made up of the remaining one hundred and thirty-eight students (66%), one hundred and ten of them being women (36.7%) and twenty-eight being men (9.3%). This random assignment of groups was performed prior to data collection and using an intact classroom procedure.

Following the recommendations of Campbell and Fiske (1959), Campbell and Stanley (1966), Pedhazur and Schmelkin (1991) and Stuart and Rubin (2008), to guarantee the internal validity of the study, we used random assignment of groups experimental/control (already mentioned) and repeated verification of the presence/absence of the treatment (predictive validity). The group equivalence was preserved by using homogeneity (χ^2^)/homoscedasticity (Levene) tests and the effectiveness of the treatment in post-test measurements was guaranteed with statistics contrast effect (*U* de Mann Whitney test, analysis of the differences in the percentages of change pre/post-test, and effect size calculations). The results of χ^2^ led us to assume the homogeneity of the samples (group by gender χ^2^ = 0.824, *p* = 0.364); the Kolmogorov Smirnov test, that there is no normality (neither in creativity nor in life satisfaction pre/post-test, by group and gender) (see Table 1), and Levene’s test that there is not homoscedasticity in CREA C pre-test and CREA A and B post-test by group, nor in CREA C pre-test by gender (see Table 2), which forced us to use non-parametric methods. To ensure external validity, we try to preserve ecological validity by performing the experiment in a situation similar to that in which the results will be generalized.

**TABLE 1:** Kolmogorov Smirnov test for creativity and life satisfaction (pre/post-test), by group and gender.

	Creativity						Life satisfaction			
	CREA A		CREA B		CREA C		SWLS		OLS	
Grupo	*Z*	*p*	*Z*	*p*	*Z*	*P*	*Z*	*p*	*Z*	*P*
Control	0.155	0.000*	0.129	0.000*	0.21	0.000*	0.218	0.000*	0.156	0.000*
Experimental	0.174	0.000*	0.168	0.000*	0.175	0.000*	0.21	0.000*	0.124	0.000*
**Género**										
Mujer	0.159	0.000*	0.138	0.000*	0.192	0.000*	0.112	0.000*	0.222	0.000*
Hombre	0.19	0.000*	0.151	0.001*	0.163	0.000*	0.141	0.002*	0.187	0.000*

CREA A, B, C = A, B, C CREA test pictures; SWLS (Satisfaction with Life Scale); OLS (Overall Life Satisfaction); c. Corrección de significación de Lilliefors; *p* = Statistical significance, *p < 0.005.

**TABLE 2:** Levene’s test for creativity and life satisfaction (pre/post-test), by group and gender.

	Group				Gender			
	*Levene’s test*	*df1*	*df2*	*p*	*Levene’s test*	*df1*	*df2*	*p*
**Creativity**								

**Pre-test**								
CREA A	2.132	1	298	0.145	0.071	1	298	0.789
CREA B	0.055	1	298	0.814	0.061	1	298	0.805
CREA C	5.392	1	298	0.021*	5.957	1	298	0.015*
**Post-test**								
CREA A	3.934	1	298	0.048*	1.041	1	298	0.309
CREA B	5.352	1	298	0.021*	0.409	1	298	0.523
CREA C	0.807	1	298	0.370	0.017	1	298	0.895

**Life satisfaction**								

**Pre-test**								
OLS	0.016	1	298	0.900	1.801	1	298	0.181
SWLS	0.054	1	298	0.816	1.343	1	298	0.247
**Post-test**								
OLS	0.061	1	298	0.805	0.611	1	298	0.435
SWLS	2.889	1	298	0.09	2.527	1	298	0.113

CREA A, B, C = A, B, C CREA test pictures; SWLS (Satisfaction with Life Scale); OLS (Overall Life Satisfaction), *p* = Statistical significance, *p < 0.005.

### Design and Procedure

The study employed was a quasi-experimental design with repeated pre-test/post-test measures and a non-equivalent control group (Cook and Campbell, 1979, 1986; León and García-Celay, 2015).

The procedure followed was: (1) inform the participants of the research objectives and request their voluntary collaboration; (2) with those who agreed to collaborate and had communicated their informed consent, we carried out the pre-test evaluation, in a class session lasting approximately one hour; (3) the intervention program was carried out in the experimental groups while the control groups received the regular institutional program; (4) after the intervention, the post-test evaluation was carried out, in which the same instruments as at pre- test were administered to both groups (both groups were administered both instruments at pre-test and post-test); (5) finally, the data was tabulated and statistically treated.

Combined with informed consent, both anonymity and the confidentiality of responses were always guaranteed (Punch, 1986; Meo, 2010; Miguélez, 2016), as well as the ethical values required in research. We respected the ethical principles and guidelines for research involving human subjects.

### The Intervention Program

The intervention program was developed in the second semester (February-June) of the 2017/18 academic year, on a similar dose to all students, in normal classroom conditions, during school hours, and incorporated in the program of each participating subject’s degree in the fields of Education and Health.

The objective of the program was to develop creative thinking and the subjective well-being of the students. The experimental conditions were performed in 8 groups with 18-23 students in each group. Each group was given on an average 7 (between 7-8) sessions (4 about life-satisfaction and 3 about creativity) of 60 min each in which 30 different activities were performed (17 of positive emotional education and 13 of creativity) (see Appendix for more information) during four months. We used different group dynamic techniques such as brainstorming, cooperative learning, role-playing, pair work, image association, information search, critical reflection, debate, case study, emotional fans, emotional self-awareness, communication, emotional regulation, empathy, etc.). The sessions were structured as follows: statement of objectives, development, and evaluation of the activity in terms of experience and learning.

The implementation of the program followed these steps: (a) previous training (first semester) of the six participating teachers in creative techniques and positive emotional management; (b) cooperative work sessions for the design of the tasks and techniques to be developed in the classroom; (c) sessions for consensus and selection of the activities to be adapted to the classroom and subject matter within the syllabus (second semester); (d) follow-up sessions and (e) evaluation sessions.

### Assessment Instruments

Creativity was measured using CREA test by Corbalán et al. (2003). It is a cognitive measure of creativity, of an individual or collective. It is based on the generation of written questions during 4 min from 3 types of visual stimuli (pictures): picture A (advisable from 10 years old to adults), picture B (advisable from 12 years old to adxults), and picture C (advisable from 6-16 years old to adults). It is used in children (from 6-11 years old), adolescents (from 12-16 years old), and adults (from age 16 years old), and has pre-existing tables of results for populations in Spain and Argentina. Unlike other tests that measure the same construct, this test identifies people’s creative potential rather than achievements and evaluates the creative process according to theories such as those of Beghetto and Kaufman (2007, cited by Corbalán and Limiñana Gras, 2010). The amount of creative production generated by individuals is explained in terms of cognitive flexibility. In that versatility in the use of cognitive schemas (Arrieta Arias, 2019), observable through the Socratic method of question generation, the authors of the test see an indicator of creative talent and a measure of original and effective creativity. Far from attempting to directly assess creative behavior as traditionally done, the CREA test places the subject in the opposite situation to the one he or she usually encounters in everyday life: asking questions, rather than giving answers, as well as in different cultures, cities, and countries. This test offers single overall creativity data (direct score) that is compared with a centile score and interpreted as high (75-99), medium (26-74), or low (1-25) creativity and has demonstrated to be a predictor of the traditionally used dimensions of creativity (Corbalán and Limiñana Gras, 2010). It has a reliability of 0.875, in the use of model A and model B as parallel models, and has concurrent validity of 0.792, when comparing model A with the Guilford Creativity Battery. It meets, therefore, the basic standards of reliability and validity for the psychometric assessment of creativity (Corbalán et al., 2003; Martínez Zaragoza, 2003; Limiñana-Gras and Javier, 2010; Elisondo, 2015). In our study, the Kendall’s coefficient of Concordance among rater’s value achieved was close to strong (Schmidt, 1997) (*W* = 0.681) and the Cronbach’s α value was: CREA A, 0.80; CREA B, 0.77 and CREA C, 0.81), and 0.90 for the test set. All these values are between 0.70 and 0.90 and indicate good internal consistency (Nunnally, 1978; Murphy and Davidshofer, 2005; Oviedo and Arias, 2005; Kaplan and Saccuzzo, 2009).

Life satisfaction was measured using the life satisfaction scale (SWLS) of Diener et al. (1985) in its Spanish version (Vázquez et al., 2013), and the single-item measure of life satisfaction, Overall Life Satisfaction Scale (OLS) of Campbell et al. (1976). SWLS is a self-evaluation scale that assesses cognitive aspects of well-being; specifically, the degree of satisfaction that the person perceives overall in his or her life. It can be used with both young people and adults (Pavot et al., 1991). The person examines the tangible aspects of his or her life, weighs the good against the bad, compares it with a standard or criterion chosen by him or her, and arrives at a judgment about one’s satisfaction with his or her life. It consists of 5 items, which are easy to understand and easy and quick to respond to. The response possibilities range from 1 to 7, where 1 is that they completely disagree and 7 is that they completely agree. The total score on the scale ranges from a minimum of 5 points to a maximum of 35 points. For this research the interpretation normative data proposed by Pavot and Diener (1993) were used: 31-35, very high score; very or highly satisfied people; 26-30, high score, satisfied people; 21-25, average score, people a little satisfied; 20, neutral; 15-19, slightly below average, a little unsatisfied; 10-14, dissatisfied, and 5-9, very or extremely dissatisfied. The Spanish version presents satisfactory psychometric properties in a sample of university students (Borrero et al., 2010). The internal consistency of the scale is adequate (Cronbach’s α = 0.84) and like that obtained in other studies: Atienza et al. (2000), α = 0.84; Reig et al. (2001), α = 0.83 and Cabañero et al. (2004), α = 0.82. In our research we obtained a Cronbach’s α value of.81, remarkably similar to reference studies, indicating good internal consistency (Nunnally, 1978; Murphy and Davidshofer, 2005; Oviedo and Arias, 2005; Kaplan and Saccuzzo, 2009).

The OLS is a single-item scale that assesses overall life satisfaction through the question: To what extent are you satisfied with your life in general? and has a Likert-type response scale of 0-10 response alternatives where 0 means completely dissatisfied and 10 means completely satisfied. It presents adequate convergent validity with other measures of satisfaction in students, such as the SLSS, the BMSLSS, and the PWI-SC, with correlation coefficients ranging from *r* = 0.50 to *r* = 0.62 for Chilean, Brazilian, Spanish, and Romanian students (Casas et al., 2015).

### Analysis of Data

We carried out a descriptive analysis of the sociodemographic variables, creativity, and life satisfaction (frequencies, percentages, means, and standard deviations), to discover the characteristics of the sample and the levels of these variables in the students of our sample before the intervention (pre-test), as well as we have calculated the medians of the variables studied to estimate Levene’s values (see Table 3).

**TABLE 3:** Descriptive analysis’s creativity and life satisfaction (pre/post-test), by group and gender.

	Creatividad/Life satisfaction	Control			Experimental			Female			Male		
		*M*	*SD*	*Me*	*M*	*SD*	*Me*	*M*	*SD*	*Me*	*M*	*SD*	*Me*
**Pre-test**	CREA A	42.29	25.67	40	32.95	25.01	25	37.34	25.63	35	36.91	26.12	25
	CREA B	48.55	26.9	50	41.05	27.14	35	46.13	26.98	45	38.94	27.61	30
	CREA C	73.16	25.78	80	65.09	27.67	75	70.4	25.818	80	63.35	30.58	75
	OLS	7.45	1.53	8	7.75	1.46	8	7.65	1.46	8	7.47	1.62	8
	SWLS	5.30,	1.07	5.4	5.42	1	5.6	5.45	0.99	5.6	5.07	1.13	5.1
**Post-test**	CREA A	47.6	25.42	50	49.35	28.27	45	48.74	27.357	50	47.88	25.788	45
	CREA B	50.4	26.13	50	52.69	29.62	46.5	53.45	27.942	50	45.44	27.723	45
	CREA C	68.59	27.39	77.5	75.37	23.78	80	73.06	25.866	80	69.51	25.076	72
	OLS	7.62	1.47	8	7.87	1.55	8	7.79	1.48	8	7.63	1.63	8
	SWLS	5.41	0.98	5.6	5.64	1.1	6	5.6	1.01	5.8	5.32	1.27	5.8

*CREA A, B, C = A, B, C CREA test pictures; SWLS (Satisfaction with Life Scale); OLS (Overall Life Satisfaction); M = mean; SD = standard deviation; Me = median.*

Next, we studied the homogeneity of samples (Pearson’s chi-square) by group (experimental/control) and gender (men/women), and both the normal distribution (Kolmogorov-Smirnov) and homoscedasticity (Levene) of the sample, considering the condition of “greater than thirty individuals” (Haier et al., 2014). As neither normality nor homoscedasticity assumptions were met in all cases, we applied non-parametric statistics (Mann Whitney *U*) to assess the treatment effect in the different groups (experimental vs. control, and men vs. women). In the cases in which we found significant differences, we calculated the effect size or Cohen’s *d* (Cohen, 1988) to evaluate its strength or magnitude, using Cohen’s criteria as a reference: small (0.20), medium (0.50), large (0.80).

In view of our need to reduce the experimental error derived from non-randomization in sample selections, in addition to the necessity to pinpoint the treatment effects of our quasi-experiment (Badii et al., 2017), we also calculated differences in the percentages of change pre/post-test. All these analyses (percentages of change and effect size) combined with Mann Whitney *U* helped to quantify and adjust the treatment means and interpret the nature of the treatment effect on the dependent variables analyzed (post-test) for each of the groups (experimental vs. control; men vs. women).

Statistical analysis of the data was performed with IBM SPSS (version 25) statistical software for Windows; with a margin in the level of accuracy of 95% and an error level of 5% (statistical significance level of α = 0.05).

## Results

### Creativity and Life Satisfaction of the Participants

In response to the first objective, we present the creativity and life satisfaction level data of the participants. The students obtained a higher average pre-test centile score in creativity in picture C (*M* = 68.80, *SD* = 27.08), followed by picture B (*M* = 44.50, *SD* = 27.24) and picture A (*M* = 37.25, *SD* = 25.70). This indicates, according to the test scale, that they present moderate levels of creative production (medium creativity). They are not outstanding in their capacity for innovation or problem solving, although on occasion they manage to do so, they make imaginative suggestions and question how to solve the problems presented to them.

Regarding life satisfaction, the mean score of our students in OLS test was 7.61 points (*SD* = 1.50), on a scale of 1 to 10, that can be interpreted as a medium-high overall satisfaction with life. These high mean scores are maintained when asked specifically about their ideal, current living conditions and achievements, and whether they would change anything about their life experiences, obtaining in SWLS test average results of 27 points (*SD* = 1.03), that indicate -according to the scale- a high score and that students are satisfied with life. People who score in this range love their lives and feel that things are going very well for them. Despite this, they have identified areas of dissatisfaction and improvement (Diener et al., 1985).

### Effects of the Intervention Program on Creativity and Satisfaction With Life

When we study the effects of the intervention program on creativity (see Table 4) we observe that there are significant median differences by group in all the CREA test pictures (A, B, C) administered before the intervention (*U*_A_ = 8723, *p* = 0.001; *U*_B_ = 9435, *p* = 0.020; *U*_C_ = 9354, *p* = 0.015) in favor of the control group (*Me*_A_ = 40; *Me*_B_ = 50 and *Me*_C_ = 80) compared to the experimental group: (*Me*_A_ = 25, *Me*_B_ = 35 and *Me*_C_ = 45). Likewise, we found group differences in the CREA C post-test (*U*_C_ = 9074, *p* = 0.005) in favor of the experimental group (*Me* = 80) vs. the control group (*Me* = 77.5), indicating a change in creativity values from medium (pre-test) to high (post-test) and a positive effect of the emotionally positive and creative intervention in the classroom for this group in this creativity test.

**TABLE 4:** Mann-Whitney *U-*test for creativity and life satisfaction (pre/post-test), by group.

	Control/Experimental groups					
	*U de Mann-Whitney*	*W de Wilcoxon*	*Z*	*Sig. Asint. (bilateral)*	*D*	*R*
**Creativity**						

**Pre-test**						
CREA A	8723	21926	−3.288	0.001*	0.369	0.181
CREA B	9435	22638	−2.333	0.02*	0.278	0.138
CREA C	9354	22557	−2.444	0.015*	0.302	0.149
**Post-test**						
CREA A	10765	20356	−0.553	0.58	−0.065	−0.033
CREA B	10718	20309	−0.615	0.538	−0.082	−0.041
CREA C	9074	18665.5	−2.819	0.005*	−0.264	−0.131

**Life Satisfaction**						

**Pre-test**						
OLS	9936.5	19527.5	−1.71	0.087	−0.201	−0.100
SWLS	10559.5	20150.5	−0.828	0.408	−0.116	−0.058
**Post-test**						
OLS	9883.5	19474.5	−1.774	0.076	−0.166	−0.082
SWLS	9082	18673	−2.806	0.005*	−0.221	−0.110

CREA A, B, C = A, B, C CREA test pictures*;* SWLS (Satisfaction with Life Scale); OLS (Overall Life Satisfaction); *U* = Mann-Whitney test; *Z* = standardized value; *p* = Statistical significance, *p < 0.005; *d* = Cohen’s d; *r* = effect size.

In the pre-test, the mean difference in creativity by group turned out to be small in each test (*d*_A_ = 0.369, *r* = 0.181; *d*_B_ = 0.278, *r* = 0.138 and *d*_C_ = 0.302, *r* = 0.149). In the post-test, we also obtained a small mean difference in creativity (CREA C) per group (*d*_Cpt_ = 0.264, *r* = 0.131).

When we consider the creativity differences in the percentages of change pre/post-test by groups (see Table 5), the Mann Whitney *U* test calculated from the normality data indicates that there are significant median changes in all the calculations of this variable (*U*_A_ = 6476.5, *p* = 0.000; *U*_B_ = 6928, *p* = 0.000 and *U*_C_ = 5389.5, *p* = 0.000) in the pre-test). The median creativity scores of the control group (*Me*_A_ = 40; *Me*_B_ = 50 and *Me*_C_ = 80) were higher than those of the experimental group (*Me*_A_ = 25; *Me*_B_ = 35 and *Me*_C_ = 45) in all tests; while in the post-test, the highest median scores were in the CREA C test of the experimental group (*Me*_C_ = 80) vs. control group (*Me*_C_ = 77.5). This confirms that the intervention program had a more significant effect on the change in median scores than those obtained by the experimental group on the CREA C test after the intervention, when compared to the control group.

**TABLE 5:** Mann-Whitney *U*-test for percentages of change in creativity and life satisfaction (pre/post-test), by group.

	Control/Experimental groups			
	*U de Mann-Whitney*	*W de Wilcoxon*	*Z*	*Sig. Asint. (bilateral)*
**Creativity**				
% CREA A	6476.5	16067.5	−6.325	0.000*
% CREA B	6928	16519	−5.721	0.000*
% CREA C	5389.5	14980.5	−7.817	0.000*
**Life Satisfaction**				
% OLS	9867	19458	−1.755	0.079
% SWLS	9483.5	19074.5	−2.288	0.022*

CREA A, B, C = A, B, C CREA test pictures*;* SWLS (Satisfaction with Life Scale); OLS (Overall Life Satisfaction); *U* = Mann-Whitney test; *Z* = standardized value; *p* = Statistical significance, *p < 0.005.

For life satisfaction, we used non-parametric statistics based on the normality data obtained. The Mann Whitney *U* test calculated (see Table 4), did not find statistical significance (α = 0.05) in the differences of medians of this variable, neither in the experimental nor in the control group in the OLS test. However, in the SWLS test, after the intervention there was a significant difference (*U* = 9082, *p* = 0.005) in favor of the experimental group (*Me* = 6, on a scale of 1-7) compared to the control group (*Me* = 5.6), with higher life satisfaction and a small difference in the effect size of this group after the intervention as indicated by Cohen’s value (*d*_pt_ = 0.221, *r* = 0.110).

After further analysis of the differences in the percentages of change of life satisfaction means in the pre-test and post-test moments in each of the groups, the Mann Whitney *U* tests for independent samples calculations confirm the non-existence of significant differences in medians in the OLS scale, and significant differences existing between the median life satisfaction scores evaluated with the SWLS (*U* = 9483, *p* = 0.022) (see Table 5), in the experimental group after the intervention (*Me* = 6, on a scale of 1-7) compared to the control group (*Me* = 5.6). These results indicate that the emotionally positive and creative intervention in the classroom improved the results of this variable (measured with SWLS) in the students who worked with this methodology in their classroom, compared to those who worked with a traditional methodology.

### Effects of the Program on Creativity and Life Satisfaction: Differences Between Genders

When we study the differences in pre-measure and post-measure separately, the Mann Whitney *U* test, calculated from the normality data, show us (see Table 6) a significant level < 0.05 that there are median differences by gender in CREA B pre-test (*U*_Bpre–test_ = 6637, *p* = 0.046), in favor of females (*Me* = 45) compared to males (*Me* = 30), and in the post-test (*U*_Bpost_ = 6626 *p* = 0.044), also in favor of females (*Me* = 50) compared to that of males (*Me* = 45). Women’s creativity is medium and above in the post-test compared to the pre-test, although the effect size is small in both cases (*d*_Bpre_ = 0.263, *r* = 0.131; *d*_Bpt_ = 0.288, *r* = 0.142), considering the index established by Cohen (1988). These analyses cannot determine whether there were any gender differences as an effect of the program, therefore also a change analysis was performed.

**TABLE 6:** Mann-Whitney *U*-test for creativity and life satisfaction (pre/post-test), by gender.

	Females/Males					
	*U de Mann-Whitney*	*W de Wilcoxon*	*Z*	*Sig. Asint. (bilateral)*	*d*	*R*
**Creativity**						

**Pre-test**						
CREA A	7801.5	10147.5	−0.138	0.890	0.017	0.01
CREA B	6637.5	8983.5	−1.993	0.046*	0.263	0.131
CREA C	7057.5	9403.5	−1.325	0.185	0.249	0.124
**Post-test**						
CREA A	7805	10151	−0.132	0.895	0.032	0.016
CREA B	6626	8972	−2.01	0.044*	0.288	0.142
CREA C	6957.5	9303.5	−1.484	0.138	0.139	0.070

**Life Satisfaction**						

**Pre-test**						
OLS	7294.5	9640.5	−0.973	0.33	0.117	0.058
SWLS	6157	8503	−2.758	0.006*	0.358	0.176
**Post-test**						
OLS	7403	9749	−0.791	0.429	0.103	0.051
SWLS	7267	9613	−0.99	0.322	0.244	0.121

CREA A, B, C = A, B, C CREA test pictures*;* SWLS (Satisfaction with Life Scale); OLS (Overall Life Satisfaction); *U* = Mann-Whitney test; *Z* = standardized value; *p* = Statistical significance, *p < 0.005; *d* = Cohen’s d; *r* = effect size.

When we calculated the differences in the percentages of change of creativity pre/post-test by gender (see Table 7), the Mann Whitney *U* test for independent samples calculations found no significant mean differences in male and female creativity.

**TABLE 7:** Mann-Whitney *U-*test for percentages of change in creativity and life satisfaction (pre/post-test), by gender.

	Females/Males			
	*U de Mann-Whitney*	*W de Wilcoxon*	*Z*	*Sig. Asint. (bilateral)*
**Creativity**				
% CREA A	7798	34826	−0.144	0.885
% CREA B	7717.5	34745.5	−0.273	0.785
% CREA C	7581	34609	−0.494	0.622
**Life Satisfaction**				
% OLS	7594.5	34622.5	−0.485	0.628
% SWLS	7396.5	34424.5	−30.79	0.429

CREA A, B, C = A, B, C CREA test pictures*;* SWLS (Satisfaction with Life Scale); OLS (Overall Life Satisfaction); *U* = Mann-Whitney test; *Z* = standardized value; *p* = Statistical significance, *p < 0.005.

To calculate the difference in life satisfaction by gender, we also considered the results of the normality tests and calculated the Mann Whitney *U* test. In Table 6 we can see, with a α = 0.05 level, that there is statistical significance only in the pre-test scores of the SWLS questionnaire (*U* = 6157, *p* = 0.006). The life satisfaction of the students, at pre-test, was higher in females (*Me* = 5.6), than in males (*Me* = 5.1). The difference in the median effect size is small, in accordance with the critical value (*d*_Dpre_ = 0.358, *r* = 0.176) developed by Cohen (1988).

In the calculations for the differences in the percentage of change pre/post-test of this variable by gender, we did not find significant variations in either measure (OLS and SWLS). The means result of life satisfaction increased in both genders from pre-test to post-test (*M*_OLS__pre–test_ female = 7.7 vs. male = 7.5; *M*_OLSpost–test_ female = 7.8 vs. male = 7.6; *M*_SWLSpre–test_ female = 5.5 vs. male = 5.1; *M*_SWLSpost–test_ female = 5.6 vs. male = 5.3) but did not reach statistical significance indicating that they were different in men and women (see Table 7).

## Discussion and Conclusion

Our research had as objectives to describe the levels of creativity and life satisfaction of the university students in our sample, to assess the effects of an emotionally positive and creative intervention program on these variables, and to analyze possible differences by gender as a result of treatment/non-treatment.

Our data have indicated that the university students analyzed have moderate or medium creativity and high life satisfaction levels before condition treatment/no treatment, coinciding with another research results such as Reboredo-Santes et al. (2012), Velásquez (2015), Lavado Pantoja (2018) and De Ávila et al. (2019).

Without underestimating from the fact that these results are good, they indicate that there is still a long way to progress in the development of these competences. Much more if we already know from Álvarez-Santullano and De Prada Creo (2018) that university students lack high levels of professional skills such as creativity and that young Spanish university students, in comparison with Europeans, has a lower level of creativity and innovation (Secanella, 2016). Along this paper we have also discussed that life satisfaction is related to personal and social well-being, employability, and professional success. Therefore, to promote actions for the improvement of creativity and life satisfaction is, among others, a challenge for university and adult education (European Association for the Education of Adults [EAEA], 2019) in particular, and for the educational system and society in general.

We also wanted to analyze group and gender differences in creativity and life satisfaction, before and after the application of an emotionally positive and creative program integrated into regular university classroom. The results indicate relevant data with an impact on university educational practice.

On group analysis, we find that the emotionally positive and creative intervention program developed had a positive impact in favor of the experimental group in creativity (which went from medium in the pretest to high in the posttest) and life satisfaction when (which went from medium-high in the pretest to high in the posttest) compared to the control group.

There are many intervention programs that have been carried out in university classrooms with satisfactory results in creativity (Amabile, 1983; Allueva, 2004; Ma, 2006; Goda, 2019; Vally et al., 2019) and subjective well-being (Sin and Lyubomirsky, 2009; Bolier et al., 2013; Lorenzo and Matallanes, 2013; Párraga Gallardo, 2015; Marrero et al., 2016; Barrios et al., 2018) like ours. This allows us to affirm that creativity and life satisfaction skills can be learned, worked on, and be profitable for university classrooms.

In his review of the duration of these programs, Lorenzo Alegría (2017) indicated that there are short programs, from 10 to 12 h; of medium duration, from 20 to 30 h; and long programs, of two years’ duration, with heterogeneous results. Our program was developed during a semester and achieved positive results, in line with other interventions such as those carried out by Vally et al. (2019), who offered a group of students who lived in the United Arab Emirates a creativity training program that ranged over an entire semester. Following the intervention, students were able to produce a greater number of ideas of an original nature and creative products.

In our research, the effect size evidenced by pre-test and post-test differences of creativity was small. It differs from other studies pointed out by Ma (2006) in which the effect size ranged from 0.47-0.76, medium-high, and Vally et al. (2019), who achieved a medium effect size. In turn, the effect size by group achieved for life satisfaction it was also small. Our data coincide with the results of some meta-analyses such as those of Sin and Lyubomirsky (2009) who analyzed 51 interventions with a total sample of 4,266 individuals. The effect sizes ranged from 0.31 to 0.84, with 96% of effect sizes in the predicted, positive direction. The meta-analysis by Bolier et al. (2013) also evidences improvements in a total of 39 interventions analyzed, 19 of them in college students, although the effect size tends to be small, but stable over time after several months (Sin and Lyubomirsky, 2009; Lyubomirsky et al., 2011; Davis and Zautra, 2013; Baños et al., 2014; Ovejero et al., 2015).

This result is modest but sufficiently relevant given the fact that life satisfaction is considered to be one essential aspect for the all-round education of citizens (European Commission, 2016), along with other skills such as innovation, entrepreneurship, and creativity. In addition, the studied variables are critical for work in a near future (Bouchard, 2014; Powell, 2018).

Regarding gender, we hypothesized that it would make significant differences in our dependent variables and that scores would be higher on the post-test. Our results show gender differences in creativity in favor of women at post-treatment moments, with an effect size small, in tune with the data of other studies that have been carried out with university students and whose evaluation test has been the CREA A as well (Bindu and Thomas, 2006; Kaufman, 2006; Miranda, 2007; Aranguren and Irazábal, 2012; Krumm et al., 2015; Elisondo and Donolo, 2016; Chiecher et al., 2018; Gamarra-Moscoso and Flores-Mamani, 2020). Other authors have also found significant differences in favor of women, like ours, but with other assessment measures and in more specific dimensions of creativity (Lee, 2005; Donolo and Elisondo, 2007; López-Martínez and Navarro-Lozano, 2008; Limiñana Gras et al., 2010).

However, the differences by gender in creativity found, were not confirmed by the analysis of differences in percentage of change. In this sense, we are forced to affirm that, although the above is true, and we were able to verify that creativity improved in the posttest, preferably in women, our study could not confirm these differences in change by gender and their data is more aligned with those of others researchers such as Maccoby and Jacklin (1974), Wright and Stone (1998), De Zubiría et al. (2003), Chacón and Moncada (2006), Matud et al. (2007), Baer and Kaufman (2008), Limiñana Gras et al. (2010), Bermejo et al. (2013) and Ramírez et al. (2019), among others, who concluded that creative potential is not significantly influenced by the gender variable.

This situation accounts for the difficulty of finding conclusive data on gender differences in creativity and invites us to continue investigating possible explanations and determinants of the heterogeneity of these results.

Finally, in life satisfaction, we did not find significant differences by gender. The means result of life satisfaction increased in both genders from pre-test to post-test but did not reach statistical significance indicating that they were different in men and women, coinciding with the results of other previous studies such as those of Alarcón (2000), Martínez (2004) and Gadermann et al. (2012) and meta-analysis such as those of Batz-Barbarich et al. (2018).

As a conclusion, our study has shown that creativity and life satisfaction can be improved in university classrooms with intervention programs such as ours, taking into account that they are competencies demanded by companies (Bouchard, 2014) and that they can have a decisive character for achievement (Fernández-Berrocal and Ruiz Aranda, 2008; Figueiredo-Ferraz et al., 2009; Anwar et al., 2012; Belmonte, 2013; Saremi and Bahdori, 2015; Bernabé et al., 2017; Peña García et al., 2017) and, as we pointed out, to improve the employability of our students, productivity, and quality of work (Diener and Biswas-Diener, 2002; del Águila Ríos et al., 2019) and the capacity for innovation that today’s society demands of us (European Commission, 2008), in agreement with the improvement guidelines of the Bologna plan, included in the Europe 2030 strategy (Sánchez Ruiz, 2020). The review by Gilar-Corbi et al. (2018), attests to the growing interest in recent years of teaching these contents and intentions to our higher education students.

Although the results have been positive and we have been able to demonstrate that the students who received this emotionally positive and creative training program improved their creativity and satisfaction with life, regardless of gender, compared to those who were immersed in the traditional methodology, we are aware that this effect can benefit from improvement.

Its impact and strength can be increased with experimental research and larger and more representative samples; with systematized and longer duration interventions that combines creativity and life satisfaction with the development of happiness, emotional intelligence and critical thinking and promote educational changes and more consistent improvements.

Future lines of research may broaden the scope of this study and contrast their results with forms of measurement different from those employed, which consider more social and emotional aspects and not so much cognitive ones, which could deepen in differential aspects by gender, as suggested by authors such as Costa et al. (1987), Bedin and Sarriera (2015), Azpiazu (2016), considering the heterogeneity of results found in the literature.

Another line of research could analyze differences in the impact of this type of intervention by age or university degree; make comparative analyses in different academic environments, contexts, or specific situations, to deepen in the study of individual variations, and the influence of intercultural and social factors in the development of these variables, taking into account the moderating effect of culture in each of them, as mentioned by authors such as Diener and Diener (1995), Alarcón (2001), Fernández-Berrocal et al. (2001), Cuadros et al. (2008), among others, in the search for a more effective intervention.

For more than 16 years we have been defending the need for a paradigm shift in universities to work in an integrated way on social and emotional skills and employability skills such as the development of critical thinking, motivation for science, entrepreneurship, and creativity, and providing empirical evidence that this is possible and necessary for the benefits it brings.

We defend a differentiated and inclusive university education in competencies (Caballero-García, 2004, 2009) that ensures equality of opportunity, culture, and gender in the competency-based education (Caballero-García and Cifuentes, 2017; Caballero-García and Sánchez, 2018; Caballero-García et al., 2019) that people and societies need to ensure their well-being and progress. Just as we propose the generation of an educational culture and policy that involves the university curriculum in key competencies such as creativity and subjective and organizational well-being, and that transcends from the university classroom to personal, social, and working life.

## Data Availability Statement

The datasets presented in this study can be found in online repositories. The names of the repository/repositories and accession number(s) can be found below: https://repositorio.ucjc.edu/handle/20.500.12020/891.

## Ethics Statement

The studies involving human participants were reviewed and approved by Comité Permanente de Integridad Académica de la UCJC. The patients/participants provided their written informed consent to participate in this study.

## Author Contributions

Both authors listed have made a substantial, direct, and intellectual contribution to the work, and approved it for publication.

## Conflict of Interest

The authors declare that the research was conducted in the absence of any commercial or financial relationships that could be construed as a potential conflict of interest.

## Publisher’s Note

All claims expressed in this article are solely those of the authors and do not necessarily represent those of their affiliated organizations, or those of the publisher, the editors and the reviewers. Any product that may be evaluated in this article, or claim that may be made by its manufacturer, is not guaranteed or endorsed by the publisher.

## Appendix

**EPROCREU Program.** Structure, activities, and objectives.

**Table:**

Creativity (Module I)		Positive emotions (Module II)	
Name of the activity	Objective	Name of the activity	Objective
Creative solutions	Raise a problem and give it a solution from different points of view and with an innovative character.	Practicum and emotions	Reflect and share emotional experiences during the internship period.
Case studies and creative decision making	Observe behaviors in different work teams when faced with a problem related to their subject.	Emocional weekly	Observe in an introspective way everything that makes them feel happy throughout a week.
Innovate educational, organizational and functional aspects of the center.	Use concepts learned in class, apply them to a real-life case, working from the different roles implied by the hat that the student has been given at random.	Dictionary of emotions	Label emotions that have taken place throughout the day.
Innovate educational, organizational and functional aspects of the center.	Formulate a problem related to the subject and agree on creative solutions.	Box of feelings	Distribute emotions based on the subjective label provided
Innovate educational, organizational and functional aspects of the center.	Provide innovative solutions to organizational and functional problems of the educational center.	You are for me	Express and generate positive emotions in others.
We innovate in education	Improve the educational problem-solving process and ask yourself what you want to innovate.	Couples	Choose the emotions that most define us and share them in couple.
I get bored	Identify the steps of the research process by reading an article, pose research problems and come up with creative solutions using the SCAMPER technique.	Emocional range	Give positive emotional feedback to others.
Match images	Find relationships between objects, apparently very different	I empathize with your emotion	Identify and understand the emotions of others.
Creativity to undertake.	Bring out the natural skills that have value in the professional field	Role playing tutor	Put into practice assertiveness and empathy linked to professional teaching practice.
Creative problem solving	Seek creative solutions to the problems raised in order to achieve greater reasoning and emotional security toward the challenges of the subject that is applied.	Sociograma	Identify the roles within the classroom.
Vídeo presentation	Encourage creativity when applying for a tempting job offer.	Express yourself and excite me	Express positive emotions through non-verbal communication
Creative brainstorming	Expand the vision of uses of certain objects for which they were not designed, through a brainstorming, encouraging reflection and divergent thinking in students.	Happy hour	Generate a good classroom environment, while working on the subject.
Reflect and associate	Encourage reflection and association of ideas on certain key concepts	Musicalia	Assess the importance of music and the rest of the senses and how its affects my writing, my concentration and my mood.
		Cooperative history of emotions	Identify the most characteristic aspects of each of the basic emotions through stories and from cooperative work
		Information sources	Improve the processes of search and academic and scientific use of information and analyze the influence of happiness and subjective well-being in organizations.
		Today I feel…	Define how the student feels before and after class.
		How much am I worth?	Promote social and linguistic skills. Being able to detect in oneself not only what he knows, but what he knows how to do, through an introspective analysis.
